# Predictive Monitoring System for Autonomous Mobile Robots Battery Management Using the Industrial Internet of Things Technology

**DOI:** 10.3390/ma15196561

**Published:** 2022-09-21

**Authors:** Kamil Krot, Grzegorz Iskierka, Bartosz Poskart, Arkadiusz Gola

**Affiliations:** 1 Faculty of Mechanical Engineering, Wrocław University of Science and Technology, ul. Łukasiewicza 5, 50-371 Wrocław, Poland; 2 Faculty of Mechanical Engineering, Lublin University of Technology, ul. Nadbystrzycka 36, 20-618 Lublin, Poland

**Keywords:** predictive monitoring, autonomous mobile robot, AMR, IIoT, Node-RED, automation stack

## Abstract

The core of the research focuses on analyzing the discharge characteristic of a lithium NMC battery in an autonomous mobile robot, which can be used as a model to predict its future states depending on the amount of missions queued. In the presented practical example, an autonomous mobile robot is used for in-house transportation, where its missions are queued or delegated to other robots in the system depending on the robots’ predicted state of charge. The system with the implemented models has been tested in three scenarios, simulating real-life use cases, and has been examined in the context of the number of missions executed in total. The main finding of the research is that the battery discharge characteristic stays consistent regardless of the mission type or length, making it usable as a model for the predictive monitoring system, which allows for detection of obstruction of the default shortest paths for the programmed missions. The model is used to aid the maintenance department with information on any anomalies detected in the robot’s path or the behavior of the battery, making the transportation process safer and more efficient by alerting the employees to take action or delegate the excessive tasks to other robots.

## 1. Introduction

Predictive maintenance is an often adopted approach for mitigating downtime of automated production systems by monitoring the condition of parts of the system to perform maintenance when it is most cost-effective [1]. Predictive monitoring on the other hand focuses on optimizing the production in real time, based on the monitored parameters in industrial applications [2,3]. In the context of battery management, predictive monitoring is widely used in electric vehicles, where the battery state of charge data can be used to plan the optimal route of the vehicle [4] but also to optimize the health of the battery [5] and its charging process [6,7]. Predictive monitoring does not have to be limited to industrial applications. It can be similarly applied in medical [8] and business [9] fields to control the system based on the measured parameters. As Internet of Things solutions rise, more processes and workstations are being interconnected through wireless communication, allowing for the creation of flexible manufacturing systems that can adapt to the changing production demands and conditions in the plant [10]. While the flexibility and the adaptivity of such manufacturing systems allow for the process to be continuous even after some unexpected disturbances in the system, it is crucial to monitor the entire system and optimize the process based on predictions of its future state based on the current and historic data [11].

The problem of battery discharge used in different types of vehicles has been widely discussed in the recent literature. Amin et al. [12] evaluated the discharge process on the LeFePO4 battery pack under different discharge current, while Xie et al. [13] proposed the model of predictive energy management for plug-in hybrid electric vehicles, considering optimal battery depth of discharge. Intelligent data-driven prognostic methodologies for estimating the remaining useful life until the end-of-discharge in real-time for lithium-polymer batteries was presented by Eleftheroglou et al. [14]. A data-driven power management strategy for plug-in hybrid electric vehicles including optimal battery depth of discharging was proposed by Xie, Qi, and Lang [15]. The energy management tool of a power system operating in a smart grid that contains electric vehicles was provided by Viegas and da Costa [16]. This year, Guo and Shen proposed a data-model fusion method for online state of power estimation of lithium-ion batteries at high discharge rate [17]. Although the proposed methods and tools are interesting, they are usually focused for non-industrial applications. In fact, there are no available studies that present solutions that are strictly dedicated to autonomous mobile robot battery management systems. Therefore, the purpose of this research was to analyze the behavior of the battery during discharge in order to create a model for predicting its future states with a selected mission queue. The example provided in the scope of this work is based on an autonomous mobile robot MiR100, which similarly to most battery-powered devices is equipped with a battery management system [18,19] that measures voltage, temperature and current, from which parameters like state of charge, state of health, and stored charge are calculated. The data provided by the BMS can be used for predictive monitoring applications that are not only beneficial for single robots but can also be used to manage fleets consisting of multiple robots. Manufacturers of autonomous mobile robots often provide such multi-robot management systems at an additional cost and with little or no possibility of connecting them to other devices in the network. For a single MiR100 robot, even if an excessive number of missions is queued up, an alarm is never raised, nor does it automatically dock into a charging station, unless it is specifically programmed in the mission. In the case of inappropriately programmed missions, no additional supervision of the robot is offered, which may lead to waste of energy, downtime, unexpected stoppage, certain missions not being executed, among other problems, making the production process suboptimal.

As an alternative to the systems provided by the manufacturers, the authors propose an open-source, flexible approach to the problem using Node-RED, an open-source programming environment for Industrial Internet of Things applications that enables the implementation of the 4^th^ generation SCADA system [20,21], supporting a wide variety of common industrial protocols [22,23], making it adaptive to virtually all modern industrial devices. Based on the parameters provided by the mobile robot, similar functionalities are offered, like managing the mission queues of the robots in the fleet, monitoring their state with the additional possibility of detecting deviations from their typical workflow, should an anomaly in the battery discharge function occur (e.g., when the default path is obstructed) [24]. Ultimately, the proposed system could be configured to monitor and control multiple devices in the Industrial Internet of Things, including other workstations or machines to manage the entire production process. It is important to notice that such a system does not need to be confined to the industrial environment only and could be used to plan routes on a much bigger scale between different facilities [25], especially considering the rise of electric vehicles and the accelerating research of autonomous solutions for transportation vehicles.

The rest of this paper is organized as follows: Section 2 describes the process of creating a lithium NMC battery discharge model for the examined mobile robot; Section 3 presents the architecture of a predictive monitoring system utilising the created model; Section 4 shows the functionality and the algorithms implemented in the proposed system, which provide information to the maintenance personnel as well as make autonomous decisions in managing the queued missions in a multirobot system; Section 5 presents the results of exemplary real-life scenarios, where the system provides an advantage from the in-house logistics perspective; Section 6 presents the conclusion of the conducted research and provides an outlook for further development of the system.

## 2. Battery Discharge Model

A model-based approach is widely used in battery management systems of electric vehicles or mobile robots, which utilize lithium-based batteries. In most cases, the models used in the BMS rely on the assumption that the charge and discharge functions of lithium-based batteries are approximately linear in the range of 20% to 90% of capacity. As a more accurate alternative, Yu et al. [26] provide an extensive analysis of different non-linear OCV-SoC models for lithium-based batteries, which could be implemented directly in the BMS of electric vehicles. In the scope of this work, a battery discharge curve is modelled for a single mission with a fixed maximum payload (100 kg) and a default length of 140 m. The autonomous mobile robot used in the experiment (MiR100) is equipped with a single Lithium NMC battery.

**Figure 1:**
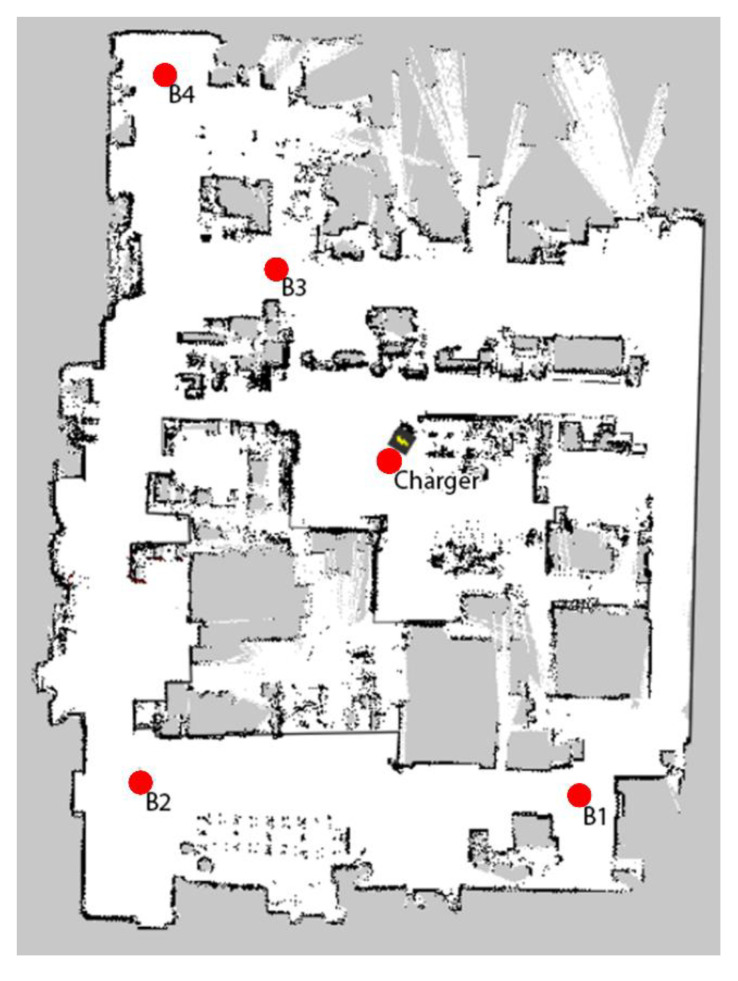
Part of the production hall layout with marked workstations to be operated by the MiR100 robot and the area where the robot can create transportation routes.

To create a reliable model of the battery discharge curve, a mission needs to be planned through the mapped area. Figure 1 presents a layout of the machine park scanned by the MiR100 robot with marked positions (B1–B4) and a charging station (Charger). The points are used to program the robot’s missions, which will provide the data necessary to model the battery discharge characteristics.

**Figure 2:**
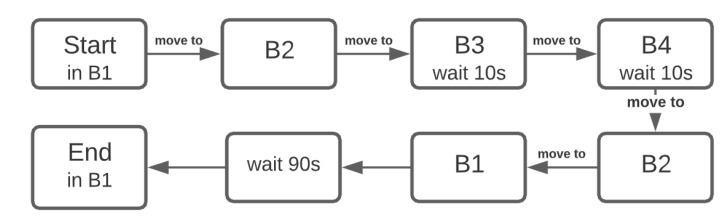
Mission algorithm used in the calibration experiment.

A mission has been planned through the points B1–B4 and has been executed with and without the obstruction of path between points B2 and B3. A simplified version of the experimental mission is presented in Figure 2.

**Figure 3:**
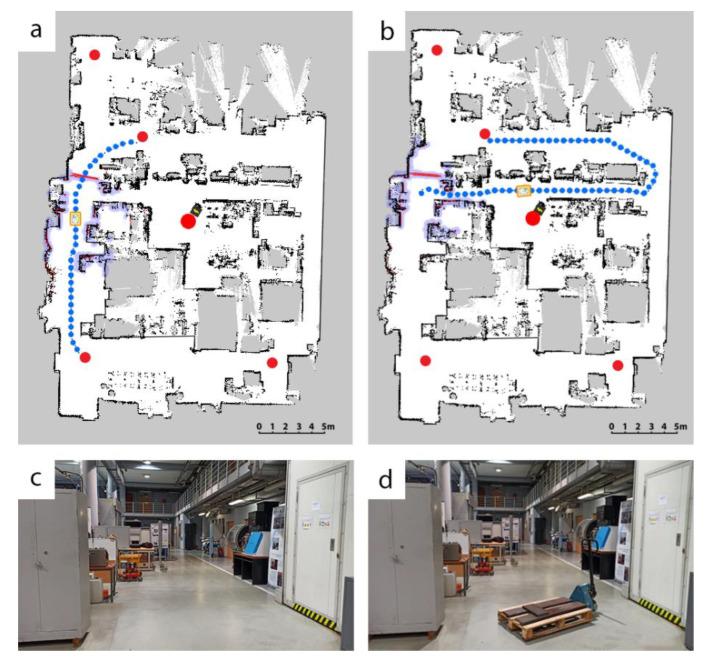
Dynamic detour planning upon detecting an obstacle: (**a**) map before detour, (**b**) planned detour upon detection of an obstacle, (**c**) unobstructed path, (**d**) obstructed path.

The autonomous robot can plan a trajectory based on the previously mapped layout visible in Figure 1 and plan a detour, should the path be obstructed as presented in Figure 3a,b. The blue dotted line shows the planned path of the robot and the red line between two red points is the obstacle detected by the robot’s LIDAR scanners. Upon reaching the obstacle, the robot tries to evade it, or if the maneuver is not possible, the robot calculates a new possible route based on the previously mapped area. The newly calculated path to point B3 is presented in Figure 3b. Figure 3c,d show the path between points B2 and B3 without and with the obstacle respectively. Such detours may cause the mission to be more energy-consuming, leading to fewer missions being executed during the entire battery discharge cycle, which in turn may cause the production process to be suboptimal.

**Figure 4:**
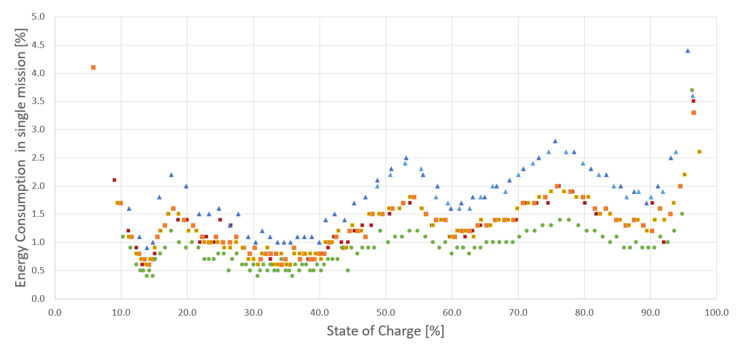
Battery discharge data for missions of different lengths.

To prevent the planned missions from not being executed, it is important to predict whether the robot will be able to execute the missions at the bottom of the stack. To model the discharge curve, a series of missions has been executed from full battery capacity down to around 10%. A single full discharge cycle takes around 10 hours with maximum payload of 100 kg, and a single lithium NMC battery (MiR100 can be equipped with up to two batteries connected in parallel). Data were collected in the range of 10–100% to see how much the values change over the entire spectrum of the battery capacity. Battery discharge data has been plotted in Figure 4 for missions of different lengths.

The calibration (◼) dataset plotted for the default mission is presented in Figure 2, and will serve as a reference for the series of measurements. Two datasets plotted for the obstacle handling (▲) present the changed energy consumption for each mission with the unplanned detour due to the obstruction of the default path. It is important to notice that the base mission was the same in these cases, and the distance travelled increased due to an unplanned detour. While the obstacle handling dataset executes longer missions, which means the energy consumption per mission is higher, to observe the characteristic of lower energy consumption per mission, additional data was gathered in a full discharge cycle for a comparative mission (●), which was shorter, less complex, and did not include an obstacle in its path. At this point in the research, the calibration data needs to be collected for each mission individually, but a more universal multivariable function $f_{mbu}$ could be determined to estimate battery usage for any of the robot’s missions.

(1) $$f_{mbu}\left(d,\;t,\;p,\;c\right)$$

where:

*d*–estimated distance to travel,
*t*–estimated time duration,
*p*–payload,
*c*–complexity of the mission.

Additional parameters may need to be included in the function $f_{mbu}$ as input variables, which may have a significant impact on the behavior of the battery SoC. While the parameters mentioned above are strictly related to a mission, the function could also include parameters related to the state of the battery itself, e.g., temperature, SoC, SoH, battery age, etc. Further research and deeper analysis need to be conducted to determine these parameters and the significance they have on the battery discharge.

The data necessary for the curve fitting can be gathered either in perfect conditions, where we can assume a correct trajectory each time, or in real-life scenarios during production processes. Assuming the perfect conditions is always difficult in real-life scenarios, especially in industrial conditions, where performing the calibration might be financially unjustifiable but the predictions can also be made based on larger datasets in real-life applications. In the scope of this publication, the calibration for each mission was conducted in controlled conditions.

To extend its uptime, the lithium NMC battery should be operating in the “battery life safe zone” rather than using the full range from 0% to 100%. Based on research [4,5,27,28], it has been proven that the optimal discharge level for batteries is between around 20% to 90%. Charging the battery to 90% and not to 100% significantly reduces the battery charging time. Therefore, the system has been designed so that the robot operates in the range of 20–90% SoC and the modelled discharge characteristic of the battery has been limited to its operating range.

The battery discharge data from the calibration dataset has been transformed into a polynomial function in Python using the NumPy’s polyfit function. To determine the degree of the polynomial, the coefficient of determination R^2^ was used for consecutive degrees. To choose the appropriate polynomial, a value of 0.9 for the coefficient of determination has been chosen as the lowest acceptable value, above which the coefficient of determination seems to stabilize, providing no significant improvement for the algorithm, but only complicating the model for larger degrees.

**Figure 5:**
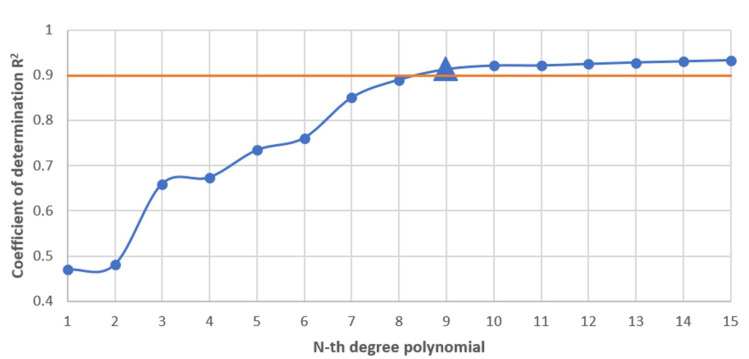
Coefficient of determination for subsequent polynomial functions of n-th degree.

As shown in Figure 5, the 9th degree polynomial meets these requirements, similarly to model 17 presented by Yu et al. [26] as the best way to model a lithium NMC battery, which is based on models presented by Xia et al. [29] and Sidhu et al. [30].

**Figure 6:**
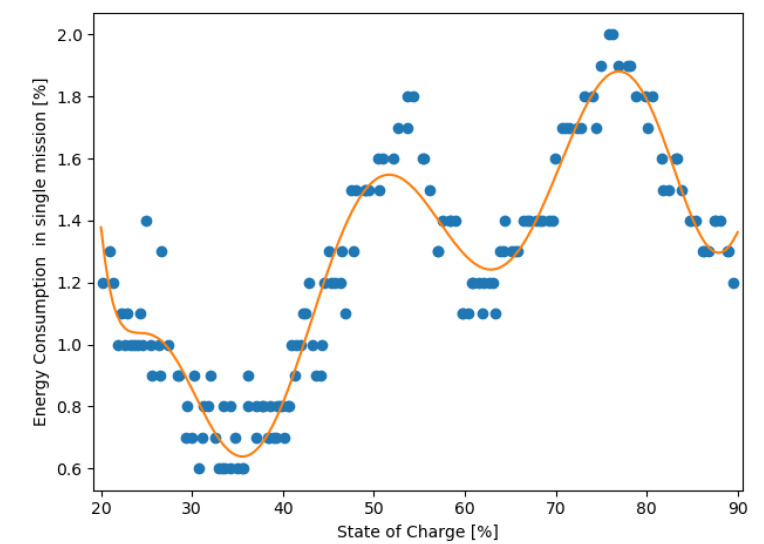
Curve fitting of the calibration dataset.

The 9th degree polynomial fitted curve has reached a coefficient of determination value of R^2^ = 0.91. Figure 6 presents the curve fitted to the dataset.

The 9th degree polynomial showing the battery discharge characteristics for the measured mission is described by Formula (2).

(2) $$y\left(x\right)=-2.88362741\times 10^{-13}x^{9}+1.48231619\times 10^{-10}x^{8}-3.28727589\times 10^{-8}x^{7}+4.11692849\times 10^{-6}x^{6}-\;3.20022662\times 10^{-4}x^{5}+1.59714909\times 10^{-2}x^{4}-5.10695739\times 10^{-1}x^{3}+1.00772980\times 10^{1}x^{2}-\;1.11378154\times 10^{2}x+5.27168295\times 10^{2}$$

where:

*y*–percentage of energy consumption in a single mission,
*x*–percentage of state of charge.

The derived formula is used in the predictive algorithm to estimate future battery usage based on the current and historic data.

## 3. Predictive Monitoring System Architecture

**Figure 7:**
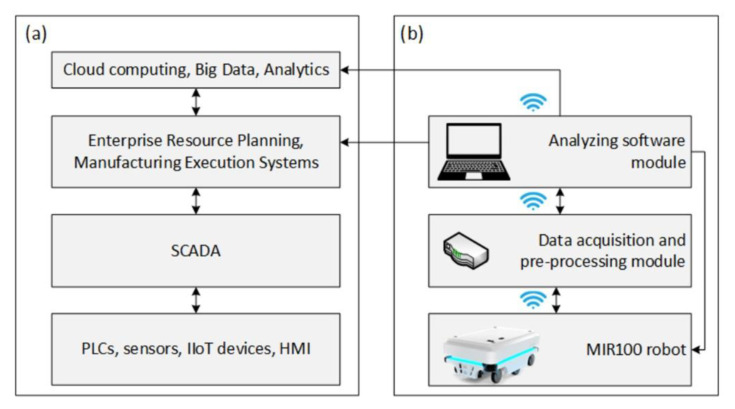
Automation stack and the predictive monitoring system: (**a**) four main levels of automation stack, (**b**) proposed predictive monitoring module for MiR100 robot.

In the course of evolution of control systems for devices used in automated manufacturing, the idea of a layered logical structure called the automation pyramid or automation stack has emerged [31], which is usually formed as a result of a practical implementation of Industry 4.0 paradigm. A typical automation stack can be presented as four main layers (Figure 7a).

The bottom layer of the stack relates to workstations, machines, and devices such as PLCs, sensors, or HMIs, equipped with communication interfaces and protocols enabling data acquisition. The second layer includes SCADA systems, which use the data to monitor and control devices at the shop floor level. These data are transferred to IT systems on the higher level, where depending on which systems have been implemented and what their functionality is, the data can be archived or used to provide feedback to the lowest level of the automation stack. Selected, key information related to production parameters are transferred to the highest level for strategic and Big Data analysis. Bearing in mind the demand for ensuring the continuity of in-house transportation, a predictive monitoring module for an autonomous mobile robot was proposed (Figure 7b).

The module presented in Figure 7b has been designed to be installed as an extension to the already implemented devices without interfering with their design or the systems included in the main automation stack. Such an approach allows the system to be integrated with multiple and various devices. In the case of the presented application, the system uses a model characteristic of the robot’s battery, but multiple characteristics or parameters could be used for different devices in the network, provided they have been modelled beforehand.

The proposed system gathers data from each executed mission, which is then used to detect anomalies in their execution. An HMI is provided to monitor the transportation processes and notify the user, should an anomaly occur. Additionally, the architecture of the predictive monitoring module resembles that of an automation stack, where the information is gathered at the bottom of the stack and is then being pre-processed, archived, and analyzed in the higher levels of the stack before being transferred to the main automation stack of the enterprise. In the case of this application, the data are provided by the robot’s BMS and recorded for every executed mission. If more missions are queued, the system estimates an overall battery consumption for the entire queue based on the battery discharge model presented in Section 2. Such information could then be used to delegate excessive missions to other robots in the fleet or alert the maintenance personnel and command the robot to recharge. The historic data can also be used by the top layers of the automation stack for further analysis and planning to optimize the production process or make it more flexible and adaptable to the changing environment.

## 4. System Implementation

The predictive monitoring system has been implemented using Node-RED, an open-source and free platform for building IIoT systems and modelling flows through an intuitive, graphical, browser-based interface, providing ready-made plugins, allowing for quick prototyping, and building highly scalable solutions. In the presented use-case, communication between the mobile robot and the predictive monitoring system was realized through an MQTT protocol and a REST API in a wireless network. A Python script has been written to simulate additional robots in the system, where the developed function was used to imitate battery usage of a real robot. The main purpose of the MiR Simulator was to tune and test the system to delegate missions between multiple robots.

The functionalities of the system are designed as consecutive steps, supporting the maintenance department with information—displaying parameters of devices connected in the system (temperature of batteries, positions of robots, mission queues, etc.), warning of any detected anomalies in the execution of missions (Yellow Alert) or the ability of the robot to execute the queued missions (Red Alert), where they are delegated to other robots in the fleet. Monitoring of additional parameters like battery temperature has been considered because of faulty batteries being used in some models of MiR robots, which has been reported by the manufacturer [32]. To monitor the battery temperature, the robot has been equipped with an external temperature sensor, providing an additional safety mechanism for the robot against potential combustion.

**Figure 8:**
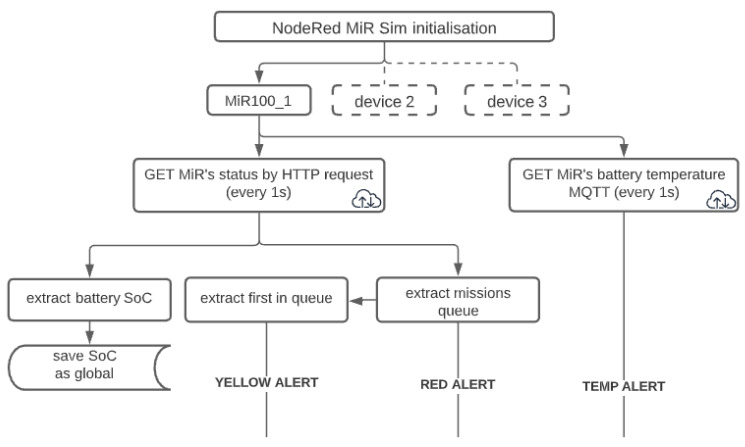
Data acquisition and pre-processing in the predictive monitoring system.

**Figure 9:**
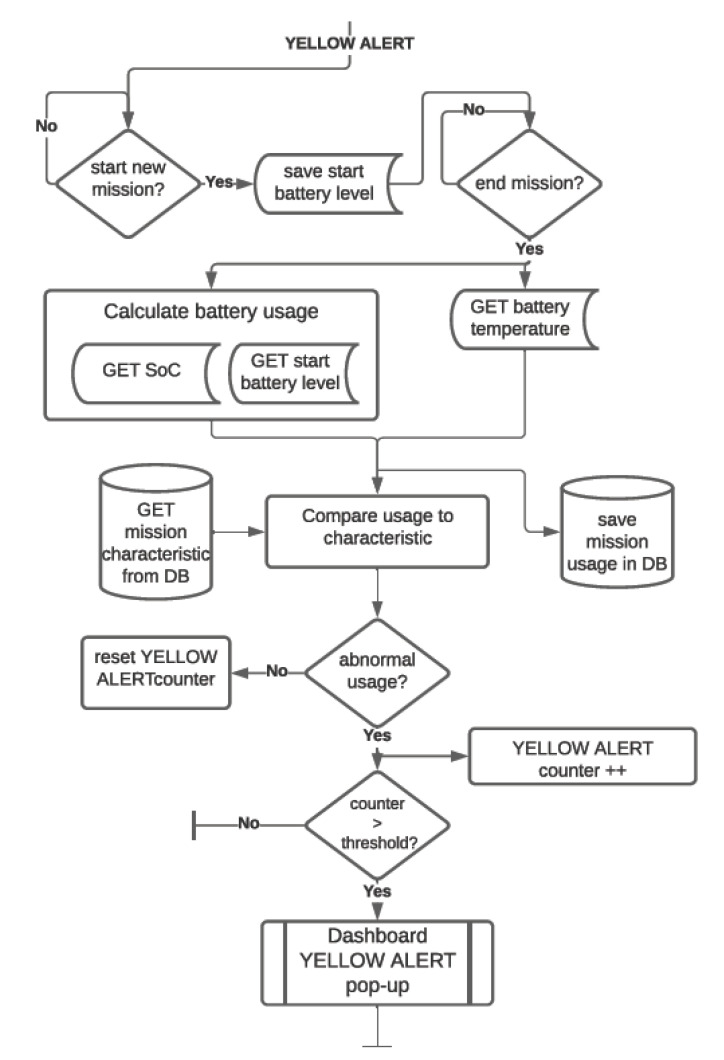
Yellow alert functionality algorithm.

**Figure 10:**
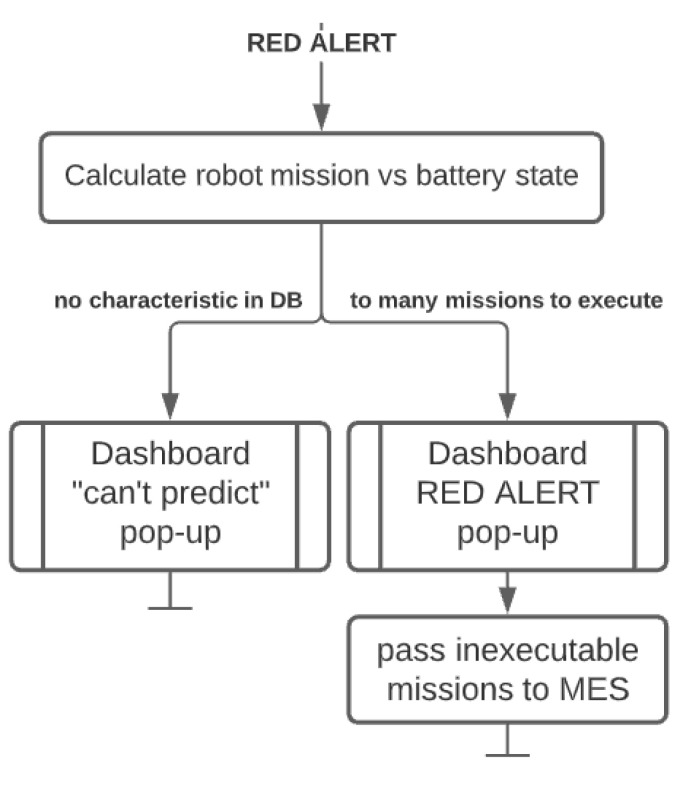
Red alert functionality algorithm.

**Figure 11:**
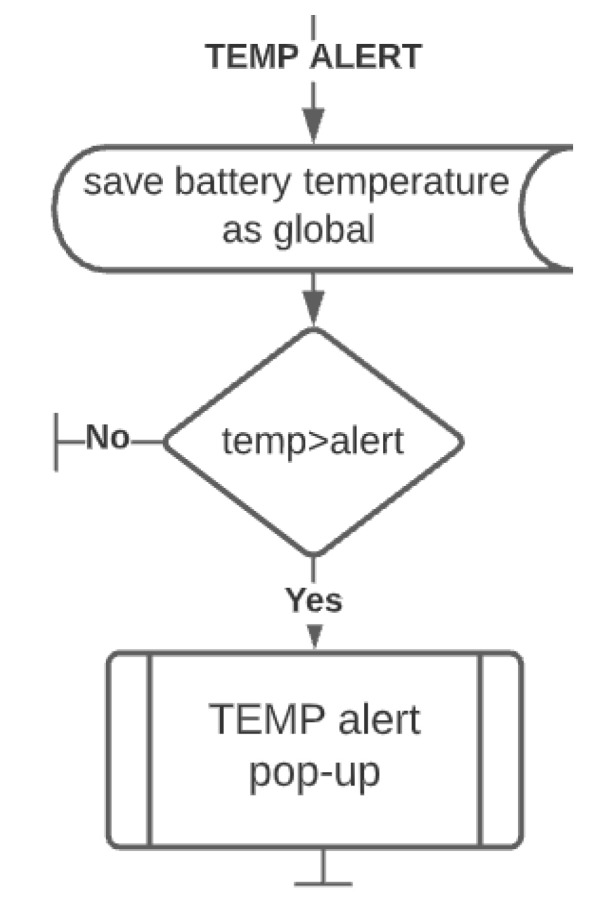
Temperature alert functionality algorithm.

More detailed algorithms of the entire system (Figure 8) and individual alerts are presented in Figure 9, Figure 10 and Figure 11 and explained below. The diagram below shows mission monitoring and management for the real robot–MiR100_1.

**YELLOW alert**: acts as an early warning for the maintenance department to check for obstacles in the regular path of the robot in case of higher-than-expected battery usage. The data gathered in the process are stored in the database for the mathematical model to be improved in further stages of research. The system allows the maintenance personnel to choose the sensitivity of the algorithm. The block diagram of the algorithm is shown in Figure 9.

**RED alert**: provides information about the queue management for a single robot, where battery consumption is estimated for the entire queue based on the battery discharge models. The system informs the maintenance personnel of any future missions unable to be executed and delegates the excessive missions to other robots in the system. In the scope of this research, the missions are delegated to the simulated robots, where they are again assessed as to whether they have enough battery to execute them. Currently, the assessment is done for the missions with a known characteristic, but a universal characteristic should be possible to derive from a bigger dataset. The block diagram of the algorithm is shown in Figure 10.

**TEMP alert**: the robot has been equipped with a temperature sensor to provide an additional monitoring feature and make the robot safer against defective, damaged, or overloaded batteries, which may cause the robot to malfunction, be damaged or even pose a fire hazard. For lithium NMC batteries exceeding 66 °C starts an exothermic chemical reaction, generating more heat. After exceeding 75 °C (without external cooling of the battery), the reaction can no longer be stopped, where the accumulation of heat and gases leads to explosion of the battery and ignition of the robot [33]. For the nominal use of the lithium NMC battery, the temperature should stay below 40 °C. The temperature warning threshold of the system has been set to 50 °C. This gives a safety buffer of at least 20 degrees before starting an uncontrolled self-ignition [34,35]. The block diagram of the algorithm is shown in Figure 11.

## 5. Experimental Verification

To verify the effectiveness of the designed predictive monitoring system with the battery discharge model, an experiment has been conducted in three scenarios:

1. unobstructed path with no changes in route,
2. obstructed path with a route changed for 16 missions due to an unexpected obstacle,
3. obstructed path with a route changed, due to an unexpected obstacle, for four consecutive missions only, due to quick reaction of the maintenance department to Yellow Alert with a fixed threshold of three consecutive missions of heightened battery usage.

The experiments have been conducted under the conditions presented in Table 1.

The following figures present the correctly (●) and incorrectly (✖) executed missions in relation to the model function derived in chapter 2 for the calibration mission (see Formula (2)). The battery usage is assumed to be incorrect for values greater than 115% of the expected battery consumption. All of the experiments were conducted in the safe range of 20% to 90% of SoC.

### 5.1. Scenario 1

In this case, the mobile robot was tasked with in-house transportation based on the experimental path described in chapter 2 without any disruptions or obstacles.

**Figure 12:**
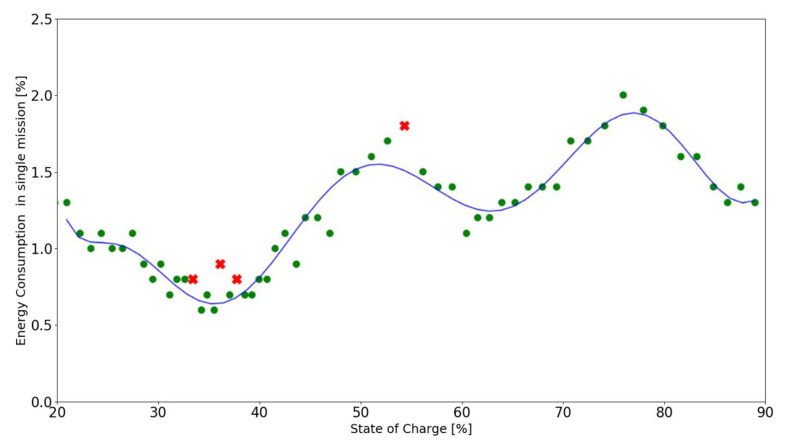
Energy consumption in experimental scenario 1, where ● represent the correctly executed missions and the ✖ represent the incorrectly executed ones.

As presented in Figure 12, the model detects four false positive results due to the fluctuation in the SoC readout from the BMS. The system functions without any disruptions, as the Yellow Alert would need three consecutive incorrectly executed missions (default threshold value in the system). In total, 58 missions were executed in this scenario.

### 5.2. Scenario 2

**Figure 13:**
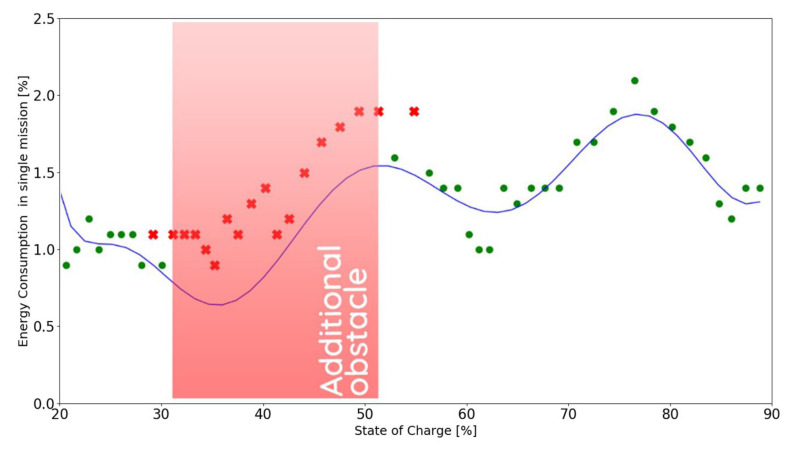
Energy consumption in experimental scenario 2, where ● represent the correctly executed missions and the ✖ represent the incorrectly executed ones.

In this scenario, an obstacle was placed in the default and shortest path for the robot for 4 hours, forcing it to make a detour, resulting in longer distance travelled and higher battery consumption per mission, which is indicated in Figure 13. In this scenario the obstacle was located in the same place as during the first investigation of the battery discharge characteristic described in chapter 2 (see Figure 3).

The addition of an obstacle resulted in the robot executing a total of 52 missions: six missions fewer than planned.

### 5.3. Scenario 3

In this scenario, the same obstacle was used; however, after three consecutive incorrectly executed missions, a Yellow Alert was raised, which prompted the maintenance personnel to investigate the situation and remove the obstacle from the path.

**Figure 14:**
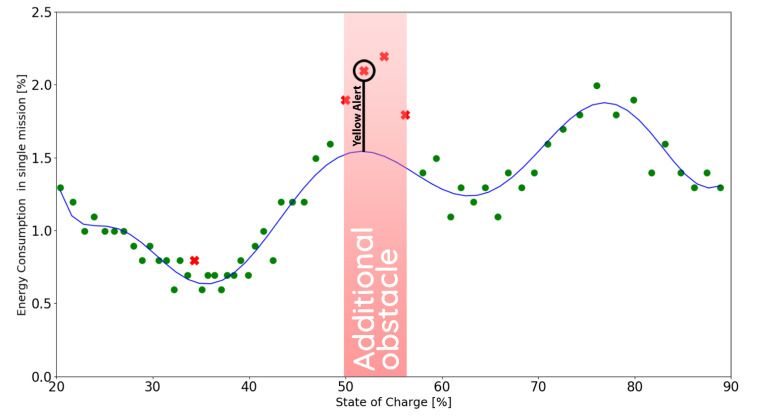
Energy consumption in experimental scenario 3, where ● represent the correctly executed missions and the ✖ represent the incorrectly executed ones.

Consequently, the robot returned to the shortest path possible for future missions as indicated in Figure 14. The quick reaction time of the maintenance personnel allowed for all of the 58 planned missions to be executed by the robot.

## 6. Discussion

Table 2 shows the comparison between the three tested scenarios, where in the case of scenario 2, no warning was communicated to the maintenance personnel, thus making the system perform much worse than in the other two scenarios, executing six fewer missions than expected.

**Figure 15:**
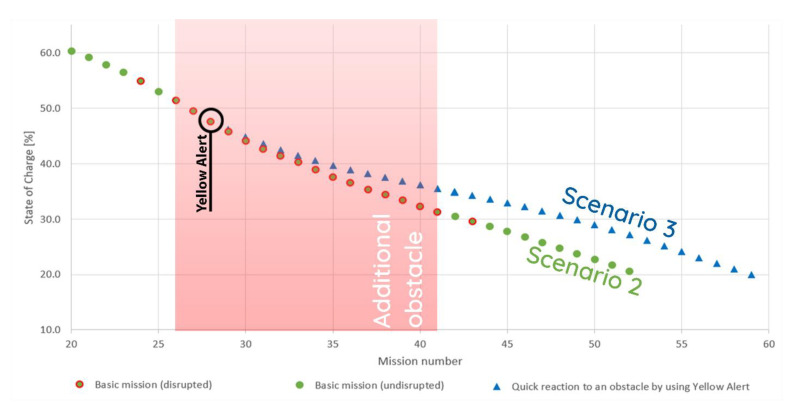
Comparison of energy consumption in experimental scenarios 2 and 3.

Upon closer inspection of scenarios 2 and 3 (see Figure 15), it becomes apparent how important the Red Alert is for the entire system. While Yellow Alert can prompt the maintenance personnel to fix any issues along the path of the robot, once their reaction time becomes too long, it is necessary to delegate any missions that the robot will not be able to complete to other robots in the fleet. In the presented case, after the additional obstacle had been removed, the system calculated that six missions would need to be delegated to other robots in the fleet because the state of charge during their execution would have dropped below 20%.

## 7. Conclusions and Outlook

In the scope of this work, the energy consumption of a battery used in a commercially available autonomous mobile robot MiR100 was analyzed. When considering lithium-based batteries, most analyses are done in the scope of voltage as a function of capacity, where the characteristic tends to be linear in most of the range. At first, the model characteristic of mission energy consumption as a function of state of charge looked unexpected, with no linearity, but the pattern was consistent across all measurements of discharge cycle. Because of this, a model characteristic of the battery discharge has been developed for a specific mission planned for the robot. The developed model characteristic was curve-fitted as a polynomial with the minimum of 0.9 R^2^, which resulted in a polynomial of 9th degree, modelling the lithium NMC battery discharge similarly to model 17 presented by Yu et al. [26].

This model characteristic has been implemented in a proposed predictive monitoring system, where it is used to determine whether the missions conducted by the connected autonomous mobile robots are executed correctly. Such information is used to inform the maintenance department of any abnormalities and to determine whether any missions need to be delegated to other robots in order to finish all of the planned missions. This data could also be used in production planning, to make the entire production process adaptable, and not only the in-house transportation.

The results of the experimental verification of the system conducted in Section 5 prove that the system helps mitigate any potential changes of the mobile robot’s route, due to early warning provided by the Yellow Alert functionality. The Red Alert functionality on the other hand allows the system to compensate for the unavoided changes in the route and the lost performance by detecting the future inability to execute some of the queued missions and delegating them to other robots in the fleet in advance.

Current approach to modelling the battery discharge curve is not universal and needs to be calibrated for every mission separately. Further investigation and experiments are necessary to find the independent variables that could define the universal function of the battery discharge, regardless of the selected mission. Potential parameters to consider as independent variables could be the number of turns, estimated travel distance, payload or battery temperature. Such a universal model would allow the robot to be truly adaptable to any disturbances in the changing environment. It would also be beneficial to investigate the discharge characteristic for batteries of different capacity, exploitation stage, or chemical composition. Additionally, in the future research, the system is planned to be expanded with wireless connection to sensors used in the stations and machines that the mobile robot interacts with to further enhance the decision making and the optimization of the entire production system.

## Figures and Tables

**Table 1:** Experimental conditions.

Robot	Payload	Route Length (Unobstructed)	Route Length (Obstructed)	Yellow Alert Threshold
MiR100	100 kg	~140 m	~200 m	3 missions

**Table 2:** Scenario comparison.

	Number of Missions		
	Nominal Battery Usage	Excessive Battery Usage	Executed in Total
**Scenario 1** **(control)**	54	4	58
**Scenario 2** **(obstacle)**	34	18	52
**Scenario 3** **(obstacle with Yellow Alert)**	53	5	58

## Data Availability

Not applicable.

## Nomenclature

AMR–Autonomous Mobile Robot

BMS–Battery Management System

IIoT–Industrial Internet of Things

OCV–Open Circuit Voltage

SoC–State of Charge

SoH–State of Health

Lithium NMC–Lithium-Nickel-Manganese-Cobalt-Oxide

HMI–Human-Machine Interface

SCADA–Supervisory Control and Data Acquisition

LIDAR–Light Detection and Ranging

MQTT–Message Queuing Telemetry Transport

PLC–Programmable Logic Controller

IT–Information Technology

REST–Representational State Transfer

API–Application Programming Interface

HTTP–Hypertext Transfer Protocol

DB–Database

MES–Manufacturing Execution System
